# Bringing Disciplines and People Together to Characterize the Plastic and Genetic Responses of Molluscs to Environmental Change

**DOI:** 10.1093/icb/icab186

**Published:** 2021-08-26

**Authors:** Omera B Matoo, Maurine Neiman

**Affiliations:** School of Biological Sciences, University of Nebraska-Lincoln, Lincoln 68588, NE, USA; Department of Biology, University of Iowa, Iowa City 52242, IA, USA; Department of Gender, Women's, and Sexuality Studies, University of Iowa, Iowa City 52242, IA, USA

## Abstract

Molluscs are remarkably diverse and are found across nearly all ecosystems, meaning that members of this ancient animal phylum provide a powerful means to study genomic-phenotype connections in a climate change framework. Recent advances in genomic sequencing technologies and genome assembly approaches finally allow the relatively cheap and tractable assembly of high-quality mollusc genome resources. After a brief review of these issues and advances, we use a case-study approach to provide some concrete examples of phenotypic plasticity and genomic adaptation in molluscs in response to environmental factors expected to be influenced by climate change. Our goal is to use molluscs as a “common currency” to demonstrate how organismal and evolutionary biologists can use natural systems to make phenotype-genotype connections in the context of changing environments. In parallel, we emphasize the critical need to collaborate and integrate findings across taxa and disciplines in order to use new data and information to advance our understanding of mollusc biology in the context of global environmental change. We end with a brief synthetic summary of the papers inspired by the 2021 SICB Symposium “Genomic Perspectives in Comparative Physiology of Molluscs: Integration across Disciplines”.

## Introduction

Mollusca is the second largest Metazoa phylum, representing over 90,000 extant taxa (Rosenberg 2014). Molluscs are found in nearly all aquatic, marine, and terrestrial habitats and harbor remarkable diversity, from octopi, snails, and oysters to the superficially wormlike Aplacophora and Polyplacophora. Originating over 500 million years ago in the Cambrian, molluscs play important ecological, economic, and medical roles across the globe (Rosenberg 2014; Fortunato 2015). Molluscs act as ecosystem engineers by introducing complexity and heterogeneity into their environments, cycling and storing carbon and nutrients, acting as biological filters in estuaries, and stabilizing the shoreline (Coen and Grizzle 2007; Commito et al. 2008). Molluscs also process and sequester calcium in their shells, bringing about habitat transformation by affecting population-, community-, and ecosystem-level processes. The bodies and shells of molluscs provide habitat structure and food resources and modify abiotic conditions (Coen and Grizzle 2007; Commito et al. 2008). Mollusc shells can also persist centuries or more after the mollusc itself has died, producing long lasting eco-historical legacies (Schöne and Surge 2005; Fortunato 2015).

Throughout human history, molluscs have served as a food source and were used across cultures and socio-economic contexts for tools, decoration, the souvenir industry, and currency exchange (Maurer 2006; Çakirlar 2011). Many mollusc species are cultivated and harvested, constituting up to 58.8% of the combined production of aquaculture and ca. 7% of capture fisheries worldwide (Darrigran et al. 2020; Dölle and Kurzmann 2020). Some molluscs (e.g., sea hare, *Aplysia*) are used in biomedical research, while others are important agricultural pests (e.g., giant African snail, *Achatina fulica*), invasive species (e.g., zebra mussel, *Dreissena polymorpha*), or intermediate vectors of deadly human parasites (e.g., bloodfluke planorb, *Biomphalaria glabrata*) (Bridger et al. 2018; Guo et al. 2019; Dölle and Kurzmann 2020).

Molluscs have gained recent attention as model species for climate change research. Global climate change is proceeding at an unprecedented rate, with major consequences for all ecosystems. A critical question in this setting whether and how natural populations will respond to global climate change and if these responses will be rapid and adequate enough for species persistence. In general, populations respond to environmental change by one or a combination of four strategies (1) shifting their range, (2) phenotypic plasticity, (3) genetic adaptation via evolution by natural selection to new conditions, and (4) persisting in the original habitat but experiencing demographic decline or extinction (Waldvogel et al. 2020).

Their high diversity and abundance along with presence across wide latitudinal clines and preservation in the fossil record means that molluscs can serve as a powerful indicator of environmental changes in all ecosystems (Fortunato 2015). Molluscs as recorders of environmental proxies are already providing valuable information about global change in aquatic ecosystems that is facilitating conservation strategies. For example, the “Mussel Watch Program”, created by NOAA's National Centers for Coastal Ocean Science (NCCOS) in response to concerns over environmental quality of the coastal and estuarine ecosystems, is one of the most successful continuous chemical contaminant biomonitoring programs in the USA (Kimbrough et al. 2008).

**Fig. 1:**
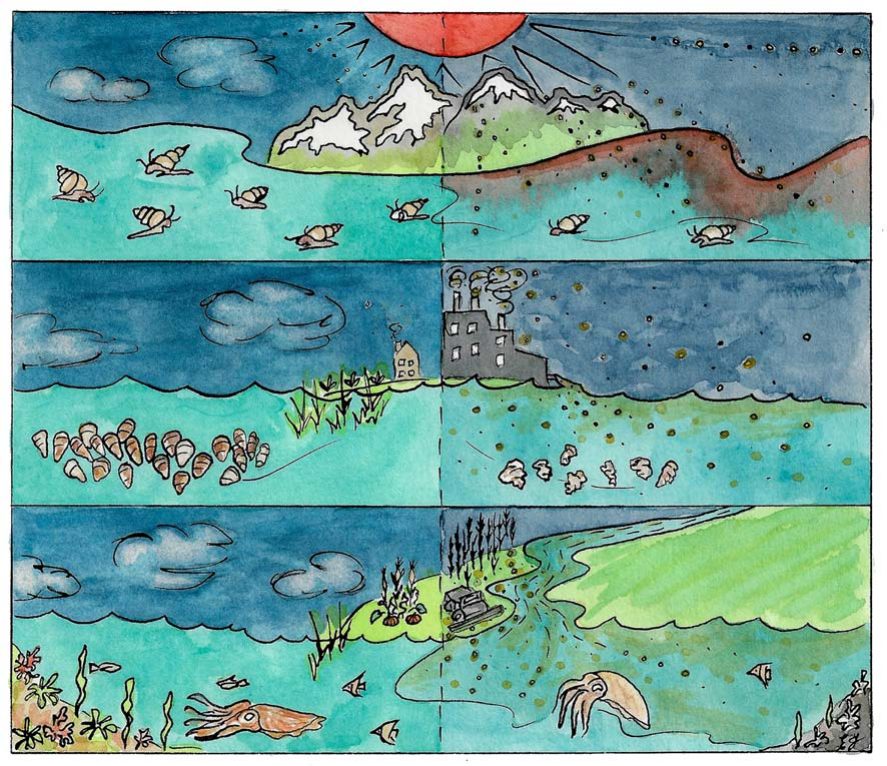
Three examples of stressors that molluscs experience as consequence of anthropogenic changes: rising temperatures (top panel), ocean acidification (middle panel), and hypoxic zones (bottom panel). Art by Emily Jalinsky.

In the following sections, we will use a case-study approach to provide some concrete examples of phenotypic plasticity and genetic adaptation in molluscs in response to global change (Fig. 1). Our survey is by no means exhaustive. Instead, our is goal is to introduce organismal and evolutionary biologists to questions of processes and patterns of organismal change, using molluscs as a “common currency”. We also discuss the need to collaborate and integrate findings across multiple disciples in order to leverage the recent availability of new DNA sequencing technologies and big genomic datasets to advance our understanding of mollusc biology in the context of global environmental change. We conclude by providing a synthesis of discussions held during the 2021 SICB Symposium “Genomic Perspectives in Comparative Physiology of Molluscs: Integration across Disciplines” alongside a summary of invited papers in this special issue.

## Molluscan responses to environmental change

As is all too common in molluscs, we know comparatively little about phenotypic plasticity and genetic variation and its association—or lack thereof—with phenotypes associated with environmental stress. Most of what we do know has come from a handful of taxa that receive relatively focused attention as model organisms. We here highlight a diverse set of Molluscan taxa that provide illustrative examples of response to various environmental stresses. It is important to note that we were unable to find a substantial body of directly relevant data (i.e., evidence for intraspecific genetic variation and/or phenotypic plasticity corresponding to differential phenotypic responses to environmental stresses) from the Molluscan classes Scaphopoda, Monoplacophora, Aplacophora, or Polyplacophora (also see Yang et al. 2020; Davison and Neiman 2021), highlighting that there is still a great deal of work left to do. Nevertheless, we believe that this overview will provide a useful starting point with respect to assessing the potential for adaptive change in molluscs in a rapidly changing world.

### Phenotypic plasticity

Phenotypic plasticity is defined as a situation when a genotype produces different phenotypes in response to different environmental conditions. Phenotypic plasticity is ubiquitous, and most traits are plastic (reviewed in Ghalambor et al. 2007). Ghalambor et al. (2007)’s review also described a wide range of consequences of plasticity with regard to organismal fitness, which in turn is a function of the particular environmental changes and the physiological limits of the organisms involved.

The specific way in which a particular genotype responds to different environments is described as a reaction norm, which can be represented by continuous or discrete character states depending on the type of trait involved (Woltereck 1909; Falconer 1990, Ghalambor et al. 2007). With respect to global change, reaction norms provide information on extant levels of phenotypic plasticity and the potential sensitivity of organisms to future global change scenarios including shifts in ecological niche breadth and resource management strategies (Ghalambor et al. 2007, 2015). Studies on phenotypic plasticity in molluscs can provide useful and actionable information regarding climate change by integrating -omic approaches. For examples, genomics-enabled studies of phenotypic plasticity can provide information about relevant genetic loci as well as transcripts that are regulated with high sensitivity for determination of physiological state (e.g., energetics, stress) and plasticity in response to environmental factors affected by climate change (e.g., pH, temperature, hypoxia) (Stillman and Armstrong 2015).

#### Case study 1: Gastropods. Nudibranchs: indicator species for tolerance–plasticity trade-off hypothesis of thermal tolerance?

Nudibranchs are soft-bodied intertidal eurythermal marine gastropods that have neither an external shell nor a water-impermeable cuticle. The absence of a shell/cuticle—in contrast to most other molluscs—and their limited mobility makes nudibranchs especially sensitive to their environment. Nudibranchs are, thus, particularly useful “indicator species” to study traits like thermal plasticity (Goddard et al. 2011; Nimbs et al. 2016; Sanford et al. 2019). A good example is provided by Armstrong et al. (2020), who investigated thermal plasticity including heat tolerance limits (CT_max_) and plasticity, temperature sensitivity of metabolism, and metabolic cost of heat shock in nine species of nudibranchs collected across a thermal gradient along the northeastern Pacific coast of California. The authors reported that adaptation to relatively warm water temperatures in intertidal nudibranchs constrains plasticity to acute thermal challenge and that southern (warm adapted) species are likely most vulnerable to future warming. Climate-related range expansions were reported during relatively warm periods (between 2014 and 2017) in more than 52 eastern Pacific nudibranch species (Goddard et al. 2018). In the southern hemisphere, an inverse southward expansion (from tropical to subtropical waters) has been reported in four tropical species of nudibranch along the Australian coast (Nimbs et al. 2015, 2016). These studies show that heat tolerance plasticity is strongly and negatively correlated with inherent heat tolerance in nudibranch molluscs. These findings are broadly in accordance with the trade-off hypothesis of thermal adaptation (Stillman 2003), which posits that organisms already adapted to high temperatures have limited scope to further increase their heat tolerance via phenotypic plasticity.

#### Case study 2: Bivalves. Oysters: strong cellular homeostasis system is a unique adaptive characteristic

The oyster is a major aquaculture species worldwide, with the highest annual production of any marine organism (http://www.fao.org). Oysters are champions of survival in the face of harsh and dynamically changing environments in estuarine and coastal zones. These habitats experience wide temporal and spatial fluctuations of temperature and salinity, and desiccation presents a serious challenge during daily and seasonal cycles. As reviewed in Zhang et al. (2016), oysters are eurythermal (range from below 0°–49°C), euryhaline (salinity tolerance below 10 parts per thousand and in excess of 35 parts per thousand), and have substantial tolerance to hypoxia and anoxia. Oysters also withstand high levels of pollutants, can concentrate metals to levels 10^3^- to 10^6^-fold higher than those of the surrounding water, and are regarded as the most useful model for studying chemical pollution in aquatic environments (reviewed in Zhang et al. 2016). Oysters have evolved remarkable phenotypic plasticity in the face of this environmental stochasticity, making these bivalves a uniquely powerful model for the study of physiological mechanisms of stress tolerance and adaptation.

Oysters have developed a wide range of sophisticated response mechanisms to maintain cellular homeostasis under stress, revealed through physiological studies (e.g., Zhang et al. 2016) and functional genomic and molecular approaches (e.g., Zhang et al. 2012). A robust homeostasis system that includes chaperone-dominated protein folding systems (HSPs and three UPR signaling pathways), xenobiotic biotransformation systems (YP450 and flavin-containing monooxygenase, glutathione S-transferases, and ATP binding cassette (ABC) transporters), and a complex antioxidant system of enzymes enables oysters to exhibit remarkable phenotypic plasticity under stress conditions (e.g., Schlenk and Buhler 1989; Boutet et al. 2004; Kingtong et al. 2007; Fabbri et al. 2008; Limon-Pacheco and Gonsebatt 2009; Zanette et al. 2011; Zhang et al. 2012; reviewed in Zhang et al. 2016). The oyster genome and transcriptome have provided a global view of the complex defense system via analysis of genome structure, gene evolution, and defenses under different stressors (Zhang et al. 2012). The expansion of key defense gene families and the strong transcriptomic response to stress highlight sophisticated genomic adaptations to sessile life in a highly stressful environment (Zhang et al. 2015).

#### Case study 3: Cephalopods. Squids: champions of hypoxia survival

Cephalopods are one of the most adaptable marine organisms, featuring a wide range of life history plasticity driven by variation in environmental conditions (Boyle and Rodhouse 2005; Hoving et al. 2013). For example, some squid taxa (e.g., Ommastrephidae) occupy oceanic niches characterized by relatively low oxygen saturation, display remarkable extremes of phenotypic plasticity, and have evolved novel physiological strategies to survive in their habitats (Seibel 2016). These species both illuminate the mechanistic process of biological regulation and forecast possible responses of marine animals to future climate change. In the last few decades, marine hypoxia has become a major ecological concern (Diaz and Rosenberg, 2008). Naturally occurring oxygen minimum zones (OMZs) with <20 μM oxygen (<10% of air saturation) constitute nearly 10% of the global ocean's volume (Paulmier and Ruiz-Pino 2009). The synergistic impact of climate-related drivers like global warming and ocean acidification further reduces oxygen availability, thereby shifting, narrowing, and compressing habitable depth and geographical ranges for many taxa (Seibel 2016).

Some squid taxa that were expected to be driven out of hypoxic areas as a consequence of anatomical and physiological constraints (e.g., *Dosidicus gigas*) instead seem to benefit from expanding hypoxia (Rosa and Seibel 2010). These squid manage to thrive under such conditions by maximizing oxygen extraction capabilities for aerobic survival in the upper ocean and by undergoing metabolic suppression during oxygen limitation at depth during the daytime (Seibel et al. 2014). In *D. gigas*, the oxygen consumption rate under 1% oxygen (P_O2_ of ∼1.0 kPa) is only ∼20% of the control rate (Rosa and Seibel 2008, 2010; Seibel et al. 2014) and metabolism (including both aerobic and anaerobic energy sources) is suppressed by ∼50% relative to controls. The remaining energy that the squid needs to thrive is generated by the activation of plastic pathways including anaerobic glycolysis and anaerobic mitochondrial metabolism that are only turned on under hypoxic conditions (Seibel 2016). Further research characterizing the molecular mechanisms facilitating this metabolic flexibility will provide an important step forward. In addition to global metabolic depression, plasticity at the transcriptional level turns on a number of hypoxia-inducible microRNAs (Hadj-Moussa et al. 2018). These microRNAs are potentially involved in cytoprotective mechanisms including neuroprotection, anti-apoptosis, regenerative mechanisms in the brain, and inhibition of apoptosis and cell proliferation, while conserving energy in the heart and limiting damage by reactive oxygen species and apoptosis in muscle (Hadj-Moussa et al. 2018).

### Genetic adaptation

Genetically based responses are the most relevant and powerful driver of long-term adaptive responses to climate change. Genomic data provides direct insight in the genetic underpinnings of these responses and is thus a central element of deciphering the mechanisms driving evolutionary adaptation to climate change (Gienapp et al. 2008; Merilä 2012).

Investigating genetic adaptation under changing environmental conditions requires knowledge of the initial (ancestral) genetic state as well as the adapted/evolved state in the new conditions (Waldvogel et al. 2020). There are two approaches commonly used to characterize the ancestral state: (1) space-for-time approach (the initial state is correlated to environmental and/or genetic heterogeneity) and (2) time-for-space approach (initial state is tracked through evolutionary time). Both of these approaches provide critical information: the former with respect to standing genetic variation, and the latter the likelihood of the evolutionary change from the known ancestral state (see Waldvogel et al. 2020 for details).

Understanding whether and how organismal populations can adapt to changing environmental conditions requires characterization of intraspecific genetic variation underlying organismal phenotypic variation in relevant environments because this variation provides the raw material for evolution by natural selection (Lewontin 1974). To what extent does genetic variation for phenotypic responses to environmental stressors exist within natural mollusc populations? This question is of critical importance both with respect to the aquaculture industry (Bernatchez et al. 2017), which plays a central role in meeting current and future food needs (Béné et al. 2016; Food and Agriculture Organization of the United Nations 2016), and in terms of predicting whether mollusc populations will be able to adapt to anthropogenic change. Broad statements regarding the genesis of intraspecific adaptive genetic diversity in molluscs will require comprehensive study of multiple members of all Mollusca classes. While inferences are limited by the narrow phylogenetic scope of available data, there is a growing body of evidence suggesting that at least some mollusc taxa feature notably high intraspecific structural variation that could in turn be adaptive (e.g., Zhang et al. 2012; Gerdol et al. 2020; Rogers et al. 2021; McElroy et al. in review).

It is less clear that nucleotide substitution will typically underlie evolutionary adaptation to environmental stresses in molluscs. While we again are limited to very cautious statements in light of the phylogenetically limited scope of relevant data, genomic data from bivalves like scallops (Wang et al. 2017) and mussels (Rogers et al. 2021) hint that structural changes like gene family expansion might ultimately be a more important source of adaptive change than single-nucleotide polymorphism at least in some taxa. Future studies should address genotype–phenotype relationships with respect to intraspecific structural polymorphisms, especially for the expanded gene families that seem to hold particular potential in contributing to stress-related adaptions in molluscs (e.g., Zhang et al. 2012, 2016; Sun et al. 2017; reviewed in Yang et al. 2020).

#### Case study 4: Gastropods. Potamopyrgus antipodarum—from pristine New Zealand lakes to invasion worldwide

These tiny New Zealand freshwater snails have risen to prominence as a model system both because of the unusual coexistence of obligately sexual and obligately asexual individuals within populations (Lively 1987) and because they are global invaders of aquatic ecosystems (Alonso and Castro-Díaz 2012). The ability to culture genetically distinct asexual lineages in the laboratory makes *P. antipodarum* an especially powerful model system with respect to identifying genetic variation for various phenotypic traits (e.g., Song et al. 2021), though the next steps of characterizing the genetic basis for this variation is much more difficult in an asexual setting.

To date, evidence for genetic variation for response to environmental stressors in *P. antipodarum* has come from studies of host-parasite interactions (e.g., Dybdahl and Lively 1995, 1998), salinity (Jacobsen and Forbes 1997), nutrient limitation (Neiman et al. 2013; Neiman and Krist 2016), temperature (Dybdahl and Kane 2005; Sharbrough et al. 2017), flow rate (Kistner and Dybdahl 2013), predation (Levri et al. 2017), and cadmium exposure (Jensen et al. 2001). These data do suggest that *P. antipodarum* can exhibit adaptive evolutionary responses to changing environments, though Verhaegen et al. (2018) suggested that a fairly minimal contribution of genetic background vs. plasticity to adaption to flow rate emphasizes plasticity over genetic variation as a potential driver of the widespread invasion of *P. antipodarum*.

#### Case study 5: Bivalves. Mytilus spp.: Can aquacultured organisms survive—and even thrive during—climate change?

These primarily saltwater mussels are found around much of the world and are of wide interest as an edible and readily aquacultured group. From a biological standpoint, various *Mytilus* taxa have risen to prominence as models for mitochondrial biology (e.g., Hoeh et al. 1991; Quesada et al. 1998; Ladoukakis and Zouros 2001; recently reviewed in Zouros and Rodakis 2019), as subject to contagious cancers (Metzger et al. 2016; Yonemitsu et al. 2019), and for elucidating the mechanisms underlying adaptation to temperature regime (e.g., Hilbish et al. 1994; Lockwood and Somero 2012).

As early as 1977, researchers demonstrated that *Mytilus edulis* exhibited genetic variation for responses to salinity (Innes and Haley 1977). Similar results with respect to salinity were subsequently reported by, for example, Bulnheim and Gosling (1988). In 2006, Freeman and Byers showed evidence consistent with a scenario whereby *M. edulis* harbors genetic variation for adaptive anti-predator responses to an invasive crab. More recent studies involving *M. edulis* have demonstrated standing adaptive genetic variation with respect to recently reported spring mortality outbreaks of unclear origin (Dégremont et al. 2019) and stresses imposed by ocean acidification (Bitter et al. 2019), and hinted that pollution might also drive adaptation (Larsson et al. 2016). In the congener *M. galloprovicialis*, Han and Dong (2020) used whole-genome sequencing data to identify adaptive genetic variation associated with environmental variables linked to heat stress. Altogether, the picture appears positive with respect to the future, though it seems imperative that aquaculturists include careful consideration of heritable variation for adaptive responses to expected environmental stresses when choosing breeding and culture stock.

#### Case study 6: Cephalopods: Does ecological opportunism and RNA editing drive recent proliferation?

Cephalopods are of wide economic and scientific interest because this ancient lineage of molluscs are often keystone species, have independently evolved sophisticated cognitive abilities, and are an important food source across the globe. Even so, no cephalopod taxon has emerged as a focus of study connecting genetic variation to phenotypes relevant to climate change. The absence of such a cephalopod model for evolutionary response to climate change might be linked to widespread challenges associated with laboratory culture, the difficulty of studying deep-water taxa, their large and often highly repetitive genomes, and the hundreds of millions of years separating cephalopods from other, better characterized animal taxa (Xavier et al. 2015; O'Brien et al. 2018; Uriarte et al. 2019).

Nevertheless, Xavier et al. (2015) argue that the persistence of coleoid cephalopods through multiple major extinction events and, more recently, surviving and even thriving despite competition with fish, has preadapted cephalopods to effectively respond to changing environments. An analogous argument was posed by Doubleday et al. (2016), who hypothesized that there might be a connection between the recent striking global increases in the abundance of many cephalopod taxa and global climate change. O'Brien et al. (2018) took this hypothesis a step further by suggesting that the remarkably extensive RNA editing discovered in cephalopods might contribute to this phenomenon. Whether these hypotheses will be supported will become clear in decades to come as climate change proceeds.

## Why a symposium on molluscs: integration across disciplines?

The revolution in DNA sequencing technologies has translated into the generation of huge bodies of data along with new genomes assembly approaches. Together, these new technologies and analytical approaches are finally allowing for the production of high-quality molluscan genome assemblies. Genome sequence data provide a particularly powerful means of linking genotype and phenotype with respect to molluscan responses (phenotypic plasticity and/adaptation) to global change. Genomic approaches can also help dissect mechanistic underpinnings by which adaptation to climate change occurs (e.g., inherited gene regulation differences by epigenetic mechanisms such as DNA methylation or histone modification; Bossdorf et al. 2008; Franks and Hoffmann 2012; via fixation of specific alleles during adaptive shifts; Hohenlohe et al. 2010).

A mechanistic understanding of the genetic basis of organismal physiology is a critically important element of forecasting whether and how organisms will respond to rapidly changing environments, which represents an urgent challenge for biologists. Our symposium used molluscs as a common currency to link biologists in otherwise quite disparate fields (e.g., biomedicine, physiology, ecology) to address genome-to-phenome research.

Newly developed genomic technologies and bioinformatic approaches have opened up new opportunities for biologists to address questions of both processes and patterns of organismal change. How can we understand the functional context of such “big” data within the intact organism, and how does genomic variation contribute to phenotype? Our symposium is especially novel from the perspective of bringing together the organismal biologists, ecophysiologists, and genomicists/bioinformaticians that are needed to provide a qualitative step forward in understanding the biology and ecology and predicting the future of one of the most important animal groups alive today. Presentations, discussions, and syntheses focused on topics including overviews of the biological and genomic diversity of molluscan life, technological progress towards highly contiguous molluscan genomes, challenges in assembling molluscan genomes, and how genome-scale processes underpin organismal physiology and interact with ecological and evolutionary processes over multiple spatiotemporal scales can be found throughout the special issue that our symposium inspired.

## Synthesis of symposium papers

This special issue presents a series of papers addressing mollusc genome-to-phenome responses in the context of global climate change. Connecting genotypes and genomic variation to functional and ecological consequences demands tools and concepts from a diverse set of fields including molecular biology, physiology, quantitative genetics, ecology, and evolutionary biology. This type of integrated approach will help to identify and decouple genetic vs. plastic underpinnings of ecologically relevant functional variation and characterize the ecological consequences of that variation. Our goal for the symposium was to bring together a transdisciplinary set of experts in mollusc biology to provide an unprecedented opportunity for knowledge exchange, discussion, and catalysis of new partnerships. The talks in our symposium featured a wide range of ecologically important concepts and traits including but not limited to immune function and symbiosis to mitochondrial performance and host-parasite interactions, and are united by their use or application of genomic techniques and resources.

Ghiselli et al. provide a comprehensive review of one of most striking features of bivalve molluscs: their peculiar mitochondrial genome biology. As Ghiselli et al. describe, bivalves have facultatively anaerobic mitochondria that allow them to survive prolonged periods of anoxia/hypoxia. Molluscs also exhibit the only known and evolutionarily stable exception to the strictly maternal inheritance of mitochondria, so-called doubly uniparental inheritance, now described in 100+ molluscan taxa to date. In this review, the authors highlight recent works studying mitochondrial biology in bivalves at the genomic and physiological level and stress that an integrated approach and collaborative relationships are the only possible ways to succeed in connecting mitochondrial genome-to-phenome relationships in bivalves.

Griffiths et al. used a single-generation selection experiment and pooled sequencing of larvae from the eastern oyster (*Crassostrea virginica*) to identify adaptive genetic variation for tolerance to low salinity in two populations from the Gulf of Mexico. The authors compared allele frequencies at 152 salinity-associated genes for larval families pre- and post-low salinity exposure and used these data to demonstrate evidence for purging of deleterious alleles at the larval stage in *C. virginica*. This study also revealed standing genetic variation for salinity tolerance and demonstrated increases in allele frequencies at multiple loci following selection, indicating a polygenic basis for adaptive responses to low salinity but also suggesting that some components of tolerance are genotype specific.

Tanner et al. measured thermal plasticity in two central California eelgrass sea hare (*Phyllaplysia taylori*) populations under four temperature-salinity scenarios in a laboratory acclimation experiment. Acclimation to warmer conditions significantly increased critical thermal minima, while low-salinity conditions resulted in high mortality. Individuals that survived the low-salinity treatments were able to respond to temperature and salinity stresses more rapidly than individuals acclimatized to saltier conditions, though the most rapid response time for the low-salinity acclimatized sea hares was at a higher temperature than the individuals acclimatized to saltier conditions. Together, these results led the authors to conclude that acclimation to climate change-induced warming will likely present challenges with respect to the ability of these sea hares to weather existing and predicted cold extremes and precipitation events.

Furr et al. explored genetic structure and physiological responses to hypoxia and immune stress challenge (the pathogen *Vibrio vulnificus*) across four populations of *Crassostrea virginica* along the North Carolina and Virginia coast. The authors observed significant genetic structure with respect to the distribution of mitochondrial cytochrome oxidase subunit I (COI) haplotypes between locations. The expression of stress-response genes including toll-like receptors, mannose receptor, defensin, and the complement gene Cq3 was specific to locations as well as to the stressors involved. Altogether, these data hint at a complex relationship between genotypes, phenotypes, and stress responses, with indirect evidence for both plasticity and genetic variation for stress responses.

Heath-Heckman and Nishiguchi used newly generated genomic sequence data from four bobtail squid taxa (*Euprymna hyllebergi, Euprymna albatrossae, Rondeletiola minor, Sepietta neglecta*), to identify regions of the genome in bobtail squids that are under selection in squid lineages that maintain symbioses with bioluminescent bacteria (all but *S. neglecta*), setting the stage to identify genes instrumental in the evolution of these mutualistic associations. This study also provided new genomic resources that will be useful for comparative work in cephalopods and beyond.

## Summary and conclusions

Our symposium and symposium papers illuminate the power and utility of molluscs as model organisms in a variety of contexts and how organismal and evolutionary biologists can leverage these fascinating organisms to generate new insights into phenotype–genotype connections. These connections are especially relevant and important in the context of anthropogenic change. We believe that we have made a strong case that molluscs can be applied to characterize the potential of and limits to plastic and evolutionary change in response to these planetary consequences of human activities. We also have emphasized the central role that cross-disciplinary collaboration and integration will play in the achievement of these goals.

## References

[bib1] Alonso A, Castro-Díez P. 2012. The exotic aquatic mud snail *Potamopyrgus antipodarum* (Hydrobiidae, Mollusca): state of the art of a worldwide invasion. Aquat Sci. 74:375–83.

[bib2] Armstrong EJ, Tanner RL, Stillman JH. 2019. High heat tolerance is negatively correlated with heat tolerance plasticity in nudibranch mollusks. Physiol Biochem Zool. 92:430–44. 31192766 10.1086/704519

[bib3] Béné C, Arthur R, Norbury H, Allison EH, Beveridge M, Bush S, Campling L, Leschen W, Little D, Squires D, et al. 2016. Contribution of fisheries and aquaculture to food security and poverty reduction: assessing the current evidence. World Dev. 79:177–96.

[bib4] Bernatchez L, Wellenreuther M, Araneda C, Ashton DT, Barth JMI, Beacham TD, Maes GE, Martinsohn JT, Miller KM, Naish KA, et al. 2017. Harnessing the power of genomics of secure the future of seafood. Trends Ecol Evol. 32:665–80. 28818341 10.1016/j.tree.2017.06.010

[bib5] Bitter MC, Kapsenberg L, Gattuso J-P, Pfister CA. 2019. Standing genetic variation fuels rapid adaptation to ocean acidification. Nat Commun. 10:5821. 31862880 10.1038/s41467-019-13767-1 PMC6925106

[bib6] Bridger JM, Brindley PJ, Knight M. 2018. The snail *Biomphalaria glabrata* as a model to interrogate the molecular basis of complex human diseases. PLoS NeglTrop Dis. 12:e0006552. 10.1371/journal.pntd.0006552 PMC6084811 30091971

[bib7] Bossdorf O, Richards CL, Pigliucci M. 2008. Epigenetics for ecologists. Ecol Lett. 11:106–15. 18021243 10.1111/j.1461-0248.2007.01130.x

[bib8] Boutet I, Tanguy A, Moraga D. 2004. Molecular identification and expression of two non-P450 enzymes, monoamine oxidase A and flavin-containing monooxygenase 2, involved in phase I of xenobiotic biotransformation in the Pacific oyster, *Crassostrea gigas*. Biochimica et Biophysica Acta (BBA) - Gene Struct Expres. 1679:29–36. 10.1016/j.bbaexp.2004.04.001 15245914

[bib85_1630703989772] Boyle P, Rodhouse P. 2005. Cephalopods: Ecology and Fisheries. Ames, Iowa: Blackwell Science Ltd.

[bib9] Bulnheim H-P, Gosling E. 1988. Population genetic structure of mussels from the Baltic Sea. HelgolÃ¤nder Meeresuntersuchungen. 42:113–29.

[bib10] Çakirlar C . 2011. Archaeomalacology Revisited: Non-dietary Use of Molluscs in Archaeological Settings. Oxford (UK): Oxbow Books.

[bib11] Coen LD, Grizzle RE. 2007. The importance of habitat created by molluscan shellfish to managed species along the Atlantic Coast of the United States. ASMFC habitat management series 8. Atlantic States Marine Fisheries Commission, Washington, District of Columbia.

[bib12] Commito JA, Como S, Grupe BM, Dow WE. 2008. Species diversity in the soft-bottom intertidal zone: biogenic structure, sediment, and macrofauna across mussel bed spatial scales. J Exp Mar Biol Ecol. 366:70–81.

[bib13] Darrigran G, Agudo-Padrón I, Baez P, Belz C, Cardoso F, Carranza A, Collado G, Correoso M, Cuezzo MG, Fabres A, et al. 2020. Non-native mollusks throughout South America: emergent patterns in an understudied continent. Biol Invas. 22:853–71.

[bib14] Davison A, Neiman M. 2021. Mobilising molluscan models and genomes in biology. Philos Trans Roy Soc B: Biol Sci. 376:20200163 (10.1098/rstb.2020.0163). PMC8059959 33813892

[bib15] Dégremont L, Maurouard E, Rabiller M, Glize P. 2019. Response to selection for increasing resistance to the spring mortality outbreaks in *Mytilus edulis* occurring in France since 2014. Aquaculture. 511:734269.

[bib87_1630704928005] Diaz RJ, Rosenberg R. 2008. Spreading dead zones and consequences for marine ecosystems. Science. 321(5891):926–9. 18703733 10.1126/science.1156401

[bib16] Dölle K, Kurzmann DE. 2020. The freshwater mollusk *Dreissena polymorpha* (zebra mussel) - a review: living, prospects and jeopardies. Asian J Environ Ecol. 13:1–17.

[bib17] Doubleday ZA, Prowse TAA, Arkhipkin A, Pierce GJ, Semmens J, Steer M, Leporati SC, Lourenço S, Quetglas A, Sauer W, et al. 2016. Global proliferation of cephalopods. Curr Biol. 26:R406–7. 27218844 10.1016/j.cub.2016.04.002

[bib18] Dybdahl MF, Kane SL. 2005. Adaptation vs. phenotypic plasticity in the success of a clonal invader. Ecology. 86:1592–601.

[bib19] Dybdahl MF, Lively CM. 1995. Diverse, endemic and polyphyletic clones in mixed populations of a freshwater snail. J Evol Biol. 8:385–98.

[bib20] Dybdahl MF, Lively CM. 1998. Host-parasite coevolution: evidence for rare advantage and time-lagged selection in a natural population. Evolution. 52:1057–66. 28565221 10.1111/j.1558-5646.1998.tb01833.x

[bib21] Fabbri E, Valbonesi P, Franzellitti S. 2008. HSP expression in bivalves. Invertebr Surviv J. 5:135–61.

[bib22] Falconer DS . 1990. Selection in different environments: effects on environmental sensitivity (reaction norm) and on mean performance. Genet Res. 56:57–70.

[bib23] Food and Agriculture Organization of the United Nations . 2016. The State of Food and Agriculture: Climate change, agriculture and food security.

[bib24] Fortunato H . 2016. Mollusks: tools in environmental and climate research. American Malacol Bull. 33:310–24.

[bib25] Franks SJ, Hoffmann AA. 2012. Genetics of climate change adaptation. Annu Rev Genet. 46:185–208. 22934640 10.1146/annurev-genet-110711-155511

[bib27] Gerdol MRM, Cruz F, Gómez-Garrido J, Vlasova A, Rosani U, Venier P, Naranjo-Ortiz MA, Murgarella M, Greco S, Balseiro P, et al. 2020. Massive gene presence-absence variation shapes an open pan-genome in the Mediterranean mussel. Genome Biol. 21:275. 33168033 10.1186/s13059-020-02180-3 PMC7653742

[bib28] Ghalambor CK, McKay J, Carroll S, Reznick D. 2007. Adaptive versus non-adaptive phenotypic plasticity and the potential for contemporary adaptation in new environments. Funct Ecol. 21:394–407.

[bib29] Ghalambor CK, Hoke KL, Ruell EW, Fisher EK, Reznick DN, Hughes KA. 2015. Non-adaptive plasticity potentiates rapid adaptive evolution of gene expression in nature. Nature. 525:372–5. 26331546 10.1038/nature15256

[bib88_1631040333892] Gienapp P, Teplitsky C, Alho JS, Mills JA, Merilä J. 2008. Climate change and evolution: disentangling environmental and genetic responses. Mol Ecol. 17(1):167–78. 18173499 10.1111/j.1365-294X.2007.03413.x

[bib30] Goddard JHR, Gosliner TM, Pearse JS. 2011. Impacts associated with the recent range shift of the aeolid nudibranch *Phidiana hiltoni* (Mollusca, Opisthobranchia) in California. Mar Biol. 158:1095–109. 24391265 10.1007/s00227-011-1633-7 PMC3873086

[bib31] Goddard JHR, Treneman N, Prestholdt T, Hoover C, Green B, Pence WE, Mason DE, Dobry P, Sones JL, Sanford E, et al. 2018. Heterobranch sea slug range shifts in the northeast Pacific Ocean associated with the 2015–16 El Niño. Proc Calif Acad Sci. 65:107–31.

[bib32] Guo Y, Zhang Y, Liu Q, Huang Y, Mao G, Yue Z, Abe EM, Li J, Wu Z, Li S, et al. 2019. A chromosomal-level genome assembly for the giant African snail *Achatina fulica*. Gigascience. 8:giz124. 31634388 10.1093/gigascience/giz124 PMC6802634

[bib33] Hadj-Moussa H, Logan SM, Seibel BA, Storey KB. 2018. Potential role for microRNA in regulating hypoxia-induced metabolic suppression in jumbo squids. Biochimica et Biophysica Acta (BBA) - Gene Regul Mech. 1861:586–93. 10.1016/j.bbagrm.2018.04.007 29729419

[bib34] Han G-D, Dong Y-W. 2020. . Anthrop Coast. 3:14–29.

[bib35] Hilbish TJ, Bayne BL, Day A. 1994. Genetics of physiological differentiation within the marine mussel *Mytilus*. Evolution. 48:267–86. 28568299 10.1111/j.1558-5646.1994.tb01311.x

[bib36] Hoeh WR, Blakley KH, Brown WM. 1991. Heteroplasmy suggests limited biparental inheritance of *Mytilus* mitochondrial DNA. Science. 251:1488–90. 1672472 10.1126/science.1672472

[bib37] Hohenlohe PA, Bassham S, Etter PD, Stiffler N, Johnson EA, Cresko WA. 2010. Population genomics of parallel adaptation in threespine stickleback using sequenced RAD tags. PLos Genet. 6:e1000862. 20195501 10.1371/journal.pgen.1000862 PMC2829049

[bib86_1630704114778] Hoving H-JT, Gilly WF, Markaida U, Benoit-Bird KJ, Brown ZW, Daniel P, Field JC, Parassenti L, Liu B, Campos B. 2013. Extreme plasticity in life-history strategy allows a migratory predator (jumbo squid) to cope with a changing climate. Glob Change Biol. 19:2089–103. 10.1111/gcb.12198 23505049

[bib38] Innes DJ, Haley LE. 1977. Genetic aspects of larval growth under reduced salinity in *Mytilus edulis*. Biol Bull. 153:312–21.

[bib39] Jacobsen R, Forbes VE. 1997. Clonal variation in life-history traits and feeding rates in the gastropod, *Potamopyrgus antipodarum*: performance across a salinity gradient. Functi Ecol. 11:260–7.

[bib40] Jensen A, Forbes VE, Parker ED Jr. 2001. Variation in cadmium uptake, feeding rate, and life-history effects in the gastropod *Potamopyrgus antipodarum*: linking toxicant effects on individuals to the population level. Environ Toxicol Chem. 20:2503–13. 11699776

[bib41] Kingtong S, Chitramvong Y, Janvilisri T. 2007. ATP-binding cassette multidrug transporters in Indian rock oyster *Saccostrea forskali* and their role in the export of an environmental organic pollutant tributyltin. Aquatic Toxicol. 85:124–32. 10.1016/j.aquatox.2007.08.006 17889379

[bib42] Kimbrough KL, Johnson WE, Lauenstein GG, Christensen JD, Apeti DA. 2008. An assessment of two decades of contaminant monitoring in the nation's coastal zone. In: NOAA technical memorandum NOS NCCOS 74. Maryland: Silver Spring.

[bib43] Kistner EJ, Dybdahl MF. 2013. Adaptive responses and invasion: the role of plasticity and evolution in snail shell morphology. Ecol Evol. 3:424–36. 23467920 10.1002/ece3.471 PMC3586651

[bib44] Ladoukakis ED, Zouros E. 2001. Direct evidence for homologous recombination in mussel (*Mytilus galloprovincialis*) mitochondrial DNA. Mol Biol Evol. 18:1168–75. 11420358 10.1093/oxfordjournals.molbev.a003904

[bib45] Larsson J, Lönn M, Lind EE, Świeżak J, Smolarz K, Grahn M. 2016. Sewage treatment plant associated genetic differentiation in the blue mussel from the Baltic Sea and Swedish west coast. PeerJ. 4:e2628. 27812424 10.7717/peerj.2628 PMC5088577

[bib46] Lewontin RC . 1974. The genetic basis of evolutionary change. New York: Columbia University Press.

[bib47] Levri EP, Landis S, Smith B, Colledge E, Metz E, Li X. 2017. Variation in predator-induced behavioral changes in introduced and native populations of the invasive New Zealand mud snail (*Potamopyrgus antipodarum Gray* 1843). Aquatic Invasions. 12:499–508.

[bib48] Limon-Pacheco J, Gonsebatt ME. 2009. The role of antioxidants and antioxidant-related enzymes in protective responses to environmentally induced oxidative stress. Mutation Research/Genetic Toxicol Environ Mutag. 674:137–47. 10.1016/j.mrgentox.2008.09.015 18955158

[bib49] Lively CM . 1987. Evidence from a New Zealand snail for the maintenance of sex by parasitism. Nature. 328:519–21.

[bib50] Lockwood BL, Somero GN. 2012. Functional determinants of temperature adaptation in enzymes of cold- versus warm-adapted mussels (Genus *Mytilus*). Mol Biol Evol. 29:3061–70. 22491035 10.1093/molbev/mss111

[bib51] Maurer B . 2006. The anthropology of money. Ann Rev Anthropol. 35:15–36.

[bib89_1631040592828] Merilä J . 2012. J. Evolution in response to climate change: in pursuit of the missing evidence. Bioessays. 34(9):811–8. 22782862 10.1002/bies.201200054

[bib53] Metzger CMJA, Luijckx P, Bento G, Mariadassou M, Ebert D. 2016. The Red Queen lives: Epistasis between linked resistance loci. Evolution. 70:480–7. 26763092 10.1111/evo.12854

[bib54] Neiman M, Krist A. 2016. Sensitivity to dietary phosphorus limitation in native vs. invasive lineages of a New Zealand freshwater snail. Ecol Appl. 26:2218–24. 27755737 10.1002/eap.1372

[bib55] Neiman M, Kay AD, Krist AC. 2013. Sensitivity to phosphorus limitation increases with ploidy level in a New Zealand snail. Evolution. 67:1511–7. 23617926 10.1111/evo.12026

[bib56] Nimbs MJ, Willan RC, Smith SDA. 2015. Range extensions for heterobranch sea slugs (formerly opisthobranch) belonging to the families Diaphanidae, Plakobranchidae and Facelinidae on the eastern coast of Australia. Marine Biodiver Reco. 8:e76.

[bib57] Nimbs MJ, Willan RC, Larkin M, Davis TR, Smith SDA. 2016. Southern range extensions for twelve heterobranch sea slugs (Gastropoda: Heterobranchia) on the eastern coast of Australia. Marine Biodiver Reco. 9:1–12.

[bib58] O'Brien CE, Roumbedakis K, Winkelmann IE. 2018. The current state of cephalopod science and perspectives on the most critical challenges ahead from three early-career researchers. Front Physiol. 9:(10.3389/fphys.2018.00700). PMC6014164 29962956

[bib59] Paulmier A, Ruiz-Pino D. 2009. Oxygen minimum zones in the modern ocean. Prog Oceanogr. 80:113–28.

[bib60] Quesada H, Gallagher C, Skibinski DAG, Skibinski DOF. 1998. Patterns of polymorphism and gene flow of gender-associated mitochondrial DNA lineages in European mussel populations. Mol Ecol. 7:1041–51.

[bib61] Rogers RL, Grizzard SL, Titus-McQuillan JE, Bockrath K, Patel S, Wares JP, Garner JT, Moore CC. 2021. Gene family amplification facilitates adaptation in freshwater unionid bivalve *Megalonaias nervosa*. Mol Ecol. 30:1155–73. 33382161 10.1111/mec.15786 PMC12066025

[bib62] Rosa R, Seibel BA. 2008. Synergistic effect of climate-related variables suggests future physiological impairment in a top oceanic predator. Proc Natl Acad Sci. 105:20776–80. 19075232 10.1073/pnas.0806886105 PMC2634909

[bib63] Rosa R, Seibel BA. 2010. Metabolic physiology of the humboldt squid, *Dosidicus gigas*: implications for vertical migration in a pronounced oxygen minimum zone. Prog Oceanogr. 86:72–80.

[bib64] Rosenberg G . 2014. A new critical estimate of named species-level diversity of the 552 recent Mollusca. American Malacol Bull. 32:308–22.

[bib65] Sanford E, Sones JL, García-Reyes M, Goddard JHR, Largier JL. 2019. Widespread shifts in the coastal biota of northern California during the 2014–2016 marine heatwaves. Sci Rep. 9:4216. 30862867 10.1038/s41598-019-40784-3 PMC6414504

[bib66] Schlenk D, Buhler DR. 1989. Xenobiotic biotransformation in the Pacific oyster (*Crassostrea gigas*). Compar Biochem Physiol Part C: Compar Pharmacol. 94:469–75. 10.1016/0742-8413(89)90100-x 2576782

[bib67] Schöne BR, Surge D. 2005. Looking back over skeletal diaries: high resolution environmental reconstructions from accretionary hard parts of aquatic organisms. Palaeogeogr Palaeoclimatol Palaeoecol. 228:1–3.

[bib68] Seibel BA, Häfker S, Trübenbach K, Zhang J, Pörtner HO, Rosa R, Storey KB. 2014. Energy metabolism during hypoxic exposure in an oxygen minimum zone squid, *Dosidicus gigas*. J Exp Biol. 217:2555–68. 24855676 10.1242/jeb.100487

[bib69] Seibel BA . 2016. Cephalopod susceptibility to asphyxiation via ocean incalescence, deoxygenation and acidification. Physiology. 31:418–29. 27708048 10.1152/physiol.00061.2015

[bib70] Sharbrough J, Cruise JL, Beetch M, Enright NM, Neiman M. 2017. Genetic variation for mitochondrial function in the New Zealand freshwater snail *Potamopyrgus antipodarum*. J Hered. 108:759–68. 28460111 10.1093/jhered/esx041

[bib71] Song Q, Magnuson R, Jalinsky J, Roseman M, Neiman M. 2021. Intraspecific genetic variation for anesthesia success in a New Zealand freshwater snail. Genetica. 149:47–54. 33389278 10.1007/s10709-020-00110-6

[bib72] Stillman JH . 2003. Acclimation capacity underlies susceptibility to climate change. Science. 301:65. 12843385 10.1126/science.1083073

[bib73] Stillman JH, Armstrong E. 2015. Genomics are transforming our understanding of responses to climate change. Bioscience. 65:237–46.

[bib74] Sun J, Zhang Y, Xu T, Zhang Y, Mu H, Zhang Y, Lan Y, Fields CJ, Ho Lam Hui J, Zhang W, et al. 2017. Adaptation to deep-sea chemosynthetic environments as revealed by mussel genomes. Nat Ecol Evol. 1:0121. 10.1038/s41559-017-0121 28812709

[bib90_1631040892204] Uriarte I, Astorga M, Navarro JC, Viana MT, Rosas C, Molinet C, Hernández J, Navarro J, Moreno-Villoslada I, Amthauer R, et al. 2019. Early life stage bottlenecks of carnivorous molluscs under captivity: a challenge for their farming and contribution to seafood production. Rev Aquacu. 11:431–57.

[bib75] Verhaegen G, McElroy KE, Bankers L, Neiman M, Haase M. 2018. Adaptive phenotypic plasticity in a clonal invader. Ecol Evol. 8:4465–83. 29760888 10.1002/ece3.4009 PMC5938463

[bib76] Waldvogel AM, Feldmeyer B, Rolshausen G, Exposito-Alonso M, Rellstab C, Kofler R, Mock T, Schmid K, Schmitt I, Bataillon T, et al. 2020. Evolutionary genomics can improve predictions of species responses to climate change. Evol Lett. 4:4–18. 32055407 10.1002/evl3.154 PMC7006467

[bib77] Wang S, Zhang J, Jiao W, Li J, Xun X, Sun Y, Guo X, Huan P, Dong B, Zhang L, et al. 2017. Scallop genome provides insights into evolution of bilaterian karyotype and development. Nat Ecol Evol. 1:0120. 10.1038/s41559-017-0120 PMC10970998 28812685

[bib78] Woltereck R . 1909. Weitere experimentelle Untersuchungen über Artver ä nderung, speziell über das Wesen quantitativer Artunterscheide by Daphniden. Verhandlungender Deutschen Zoologischen Gesellschaft. 19:110–92.

[bib79] Xavier JC, Allcock AL, Cherel Y, Lipinski MR, Pierce GJ, Rodhouse PGK, Rosa R, Shea EK, Strugnell JM, Vidal EAG, et al. 2015. Future challenges in cephalopod research. J Marine Biol Ass United Kingdom. 95:999–1015.

[bib80] Yang Z, Zhang L, Hu J, Wang J, Bao Z, Wang S. 2020. The evo-devo of molluscs: Insights from a genomic perspective. Evol Dev. 22:409–24. 32291964 10.1111/ede.12336

[bib81] Yonemitsu MA, Giersch RM, Polo-Prieto M, Hammel M, Simon A, Cremonte F, Avilés FT, Merino-Véliz N, Burioli EAV, Muttray AF, et al. 2019. A single clonal lineage of transmissible cancer identified in two marine mussel species in South America and Europe. eLife. 8:e47788. 31686650 10.7554/eLife.47788 PMC6831032

[bib82] Zanette J, Alves de Almeida E, Zaccaron da Silva A, Guzenski J, Fernando Ferreira J, Di Mascio P, Risoleta Freire Marques M, Celso Dias Bainy C. 2011. Salinity influences glutathioneS-transferase activity and lipid peroxidation responses in the *Crassostrea gigas* oyster exposed to diesel oil. Sci Total Environ. 409:1976–83. 21349572 10.1016/j.scitotenv.2011.01.048

[bib83] Zhang G, Fang X, Guo X, Li L, Luo R, Xu F, Yang P, Zhang L, Wang X, Qi H, et al. 2012. The oyster genome reveals stress adaptation and complexity of stress formation. Nature. 490:49–54. 22992520 10.1038/nature11413

[bib84] Zhang L, Li L, Guo X, Litman GW, Dishaw LJ, Zhang G. 2015. Massive expansion and functional divergence of innate immune genes in a protostome. Sci Rep. 5:8693 25732911 10.1038/srep08693 PMC4346834

[bib85] Zhang G, Li L, Meng J, Qi H, Qu T, Xu F, Zhang L. 2016. Molecular basis for adaptation of oysters to stressful marine intertidal environments. Ann Rev Anim Biosci. 4:357–81. 26515272 10.1146/annurev-animal-022114-110903

[bib86] Zouros E, Rodakis GC. 2019. Doubly uniparental inheritance of mtDNA: An unappreciated defiance of a general rule. In: Sutovsky, editor. Cellular and molecular basis of mitochondrial inheritance. Cham: Springer International Publishing. p. 25–49. 10.1007/102_2018_4 30637482

